# Nucleation in Amorphous Terfenadine at a Temperature Much Lower than the Glass Transition Temperature and Its Impact on Physical Stability

**DOI:** 10.1021/acs.molpharmaceut.5c00700

**Published:** 2025-08-25

**Authors:** Katsutoshi Yamaguchi, Yuya Ishizuka, Etsushi Yoshikawa, Takashi Makishima, Ryo Mizoguchi, Kohsaku Kawakami

**Affiliations:** † Pharmaceutical Developability, CMC Research, Astellas Pharma Inc., 21Miyukigaoka, Tsukuba, Tsukuba, Ibaraki 3058585, Japan; ‡ Analytical Research 1, Analytical Research Laboratories, CMC Development, Astellas Pharma Inc., 21 Miyukigaoka, Tsukuba, Ibaraki 3058585, Japan; § Medical Soft Matter Group, Research Center for Macromolecules and Biomaterials, National Institute for Materials Science, 1-1 Namiki, Tsukuba, Ibaraki 3050044, Japan; ∥ Graduate School of Science and Technology, University of Tsukuba, 1-1-1 Tennodai, Tsukuba, Ibaraki 3058577, Japan

**Keywords:** amorphous, nucleation, crystal growth, physical stability, local molecular mobility

## Abstract

In this study, the crystallization behavior of amorphous terfenadine (TFD) was investigated with a focus on nucleation temperature. The cold crystallization behavior of amorphous TFD annealed at various temperatures and the resultant crystal form were evaluated by using differential scanning calorimetry and powder X-ray diffraction. Samples annealed at −20 °C provided the lowest cold crystallization temperature and the highest proportion of form II, indicating that nucleation for form II was enhanced at a temperature much lower than the glass transition temperature (58 °C). Initiation time for isothermal crystallization of TFD at 100 °C was shortened by 30% by applying annealing at −20 °C beforehand. In contrast, dissolution and supersaturation behaviors were not affected by nucleation, as the crystal form of the precipitate was different from that induced by annealing. The isoconversional Kissinger–Akahira–Sunose model was applied to analyze the cold crystallization kinetics to find an increase in the apparent frequency factor for samples annealed at −20 °C, suggesting the presence of preformed crystal nuclei. Lastly, the anomalous nucleation behavior of amorphous TFD at low temperatures was discussed from the framework of classical nucleation theory and molecular mobility. This study provides important insights into the nucleation and crystal growth behaviors of amorphous pharmaceutical compounds.

## Graphical abstract

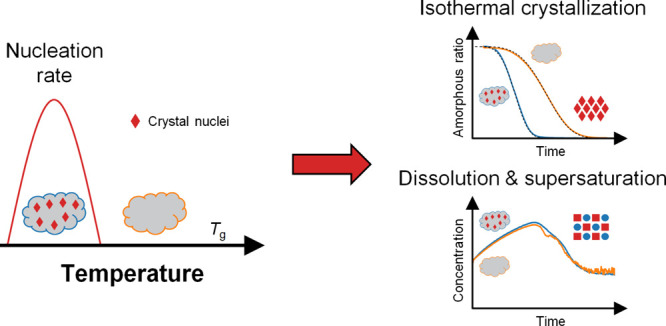

## 1 Introduction

Poor solubility of drug candidates for clinical development is a serious problem for pharmaceutical companies. Amorphization is one of the most effective approaches for improving the solubility and bioavailability of drug candidates.^1–3^ However, crystallization is detrimental to amorphous formulations because it diminishes the solubility advantage of the amorphous form. Control of the physical stability of amorphous solids is challenging because the amorphous state is thermodynamically unstable.^4,5^ Polymer excipients are generally used to stabilize amorphous formulations and improve their dissolution performance.^1,6,7^

Crystallization is a two-step process that consists of nucleation and crystal growth. The temperature dependence of these processes is governed by the balance of thermodynamic driving force, interfacial energy, and molecular mobility.^8–10^ The thermodynamic driving force toward crystallization becomes larger as temperature decreases, while molecular mobility is suppressed particularly below the glass transition temperature (*T*_g_). Generally, the nucleation rate reaches a maximum just above *T*_g_, whereas the crystal growth rate is enhanced between *T*_g_ and the melting temperature (*T*_m_).^8–12^ However, exceptional cases have been reported, wherein the nucleation rate was enhanced at a temperature much lower than *T*_g_.^13–16^ In our previous studies, nucleation in celecoxib glass (*T*_g_: 58 °C) for form III was most strongly enhanced at −50 °C.^13^ Furthermore, nucleated celecoxib glass showed diminished physical stability and dissolution performance compared to the fresh glass.^17,18^ A few hypotheses have been proposed to explain these anomalous nucleation behaviors: β relaxation, a local motion of molecules observed even below *T*_g_, is implicated in the nucleation process,^14,15^ and crack formation in amorphous solids increases molecular mobility or changes intermolecular interaction, resulting in enhanced nucleation.^16,19^ Crack formation is particularly observed when amorphous solids are prepared by quench cooling of the melt, presumably due to the mechanical stress caused in the glasses during a rapid change in volume.

The purpose of this study is to investigate nucleation behavior and to assess its impact on physical stability and dissolution of an amorphous pharmaceutical compound. We attempted to find a compound with similar nucleation behavior to celecoxib glass, in which nucleation was enhanced at a temperature much lower than *T*_g_,^13^ and selected terfenadine, TFD, (1-(4-*tert*-butylphenyl)-4-[4-[hydroxy­(diphenyl)­methyl]­piperidin-1-yl]­butan-1-ol) (Figure 1). This compound exhibited enhanced crystallization behavior after annealing at −20 °C, which is significantly lower than *T*_g_ (58 °C),^20,21^ in the screening study. TFD is a selective antagonist of the histamine H_1_ receptor, which was previously used for the treatment of histamine-mediated disorders, such as allergic rhinitis,^22^ but was withdrawn from the market due to the risk of cardiotoxicity.^23^ TFD is a racemic compound and shows crystalline polymorphism.^21,24,25^ The common polymorphs of TFD crystals are form I and form II, with form I being monotropically stable against form II.^21,24,25^ Since TFD is classified as class II or IV according to the biopharmaceutical classification system because of its low solubility,^26,27^ the amorphous form of it has been widely reported in many research articles as a model compound. Amorphous TFD is classified as class III^28^ under the classification system for the crystallization tendency of organic molecules proposed by Baird et al.^29^ That is, it does not crystallize during cooling of the melt and subsequent reheating under the specified conditions. Class III behavior is assumed to originate from significant separation between the temperatures of nucleation and crystal growth and/or lower rates of nucleation and crystal growth.^30^ Both global and local molecular mobilities must be understood well for elucidating the nucleation and crystal growth behaviors of amorphous materials. On intensive evaluation of the molecular mobility of amorphous TFD by dielectric relaxation spectroscopy and molecular dynamics simulations,^20,31^ α relaxation was detected above its *T*_g_, and γ and supplementary relaxation were observed below its *T*_g_ in the absence and presence of water molecules, respectively.

**Figure 1:**
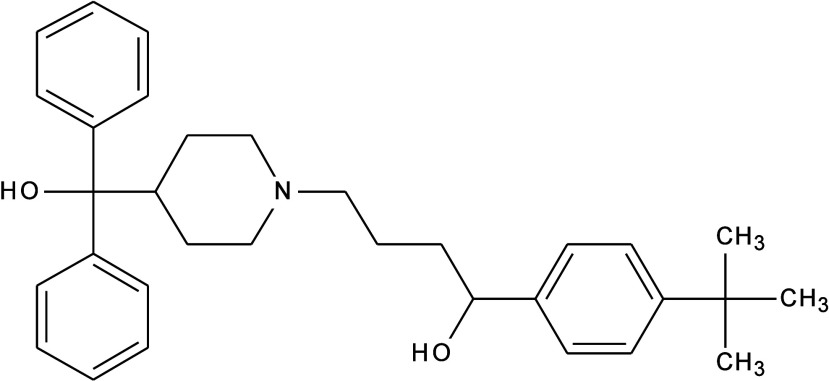
Chemical structure of TFD.

Here, we investigated the crystallization behavior of amorphous TFD using differential scanning calorimetry (DSC) and polarized light microscopy (PLM), with a focus on the nucleation temperature. We also investigated the effect of nucleation on physical stability and dissolution/supersaturation performance and the impact of crack formation on nucleation behavior. Furthermore, we attempted to explain the anomalous nucleation behavior of amorphous TFD at low temperatures in the framework of classical nucleation theory and molecular mobility.

## 2 Materials and Methods

### 2.1 Materials

Crystalline TFD powder was purchased from Sigma-Aldrich (St. Louis, MO, USA), and the crystal form was identified as form I on receipt. Japanese Pharmacopoeia second (JP2) fluid for the disintegration test, which is a phosphate buffer of pH 6.8, was obtained from Nacalai Tesque (Kyoto, Japan). Reagent-grade ethyl acetate, HPLC-grade acetonitrile, and Wako special-grade trifluoroacetic acid (TFA) were obtained from FUJIFILM Wako Pure Chemical (Osaka, Japan). Milli-Q water was generated using an ultrapure water purification system (Merck Millipore, Burlington, MA, USA). All reagents were used as received.

Form II of the TFD crystal was prepared by the following procedure. Form I of TFD crystals was dissolved in ethyl acetate at a concentration of 40 mg/mL at 70 °C and then gently stirred at 5 °C for 4 h. The residues were collected by vacuum filtration and vacuum-dried overnight at 50 °C. Thermodynamic parameters of amorphous and crystalline TFD are shown in Table 1 and agreed well with previously reported values.^20,21,24,25,28,32–37^

**Table 1:** Thermodynamic Parameters of Amorphous and Crystalline TFD^a^

thermodynamic parameter	value
*T*_g_ (°C)	58.4
Δ*C*_p_ (J/(mol·K))	271.4
*T*_m_ (°C)	149.9 (I), 145.9 (II)
Δ*H*_f_ (kJ/mol)	54.4 (I), 53.3 (II)
Δ*S*_f_ (J/(mol·K))	128.5 (I), 127.3 (II)

a *T*_g_: glass transition temperature, Δ*C*_p_: heat capacity change at *T*_g_, *T*_m_: melting temperature, Δ*H*_f_: enthalpy of fusion, Δ*S*_f_: entropy of fusion, I: form I, II: form II.

### 2.2 Preparation of Amorphous TFD

Amorphous TFD was prepared by quench cooling of the melt using a DSC Q2000 or DSC 2500 (TA Instruments, New Castle, DE, USA). 3 mg of TFD powder was weighed in a Tzero pan (TA Instruments) and sealed with a Tzero hermetic lid (TA Instruments) with a pinhole. Then, the samples were heated to 165 °C, which is approximately 15 °C above *T*_m_ at a rate of 20 °C/min, held isothermally for 1 min, and cooled to 25 °C at a rate of 20 °C/min in DSC. Amorphous TFD with intentional cracks was prepared by the following procedure: TFD melt was cooled to −20 °C at a rate of 20 °C/min, held isothermally for 10 min, and then heated to 25 °C at a rate of 10 °C/min. Subsequently, a DSC pan containing amorphous TFD was placed on an aluminum plate and tightly closed in a storage container with silica gel and then placed in a stability chamber immediately after amorphization. Samples were collected from the chambers after predetermined periods of time and used for evaluation.

All DSC instruments were calibrated using indium and sapphire and are used throughout the following sections. All DSC measurements were performed under a nitrogen gas flow of 50 mL/min, and data were recorded at 10 points/s. Thermograms were analyzed by using Universal Analysis 2000 or TRIOS software (TA Instruments).

### 2.3 DSC Measurement

Amorphous TFD, annealed under various conditions, was collected from chambers and heated from 25 to 160 °C at a rate of 1 °C/min to evaluate thermal events during heating in DSC. DSC measurements of freshly prepared amorphous TFD were performed using the same procedure without taking samples out of the DSC instrument after quench cooling of the melt. A heating rate of 10 °C/min was also used for fresh glass and for glass annealed at −20 °C for 1 day, in addition to 1 °C/min.

The apparent kinetic parameters of cold crystallization process were evaluated using the Kissinger–Akahira–Sunose (KAS) equation:^38,39^

$$\ln\left(\frac{\beta }{{T_{\alpha }}^{2}}\right)=-\frac{E_{a}}{RT_{\alpha }}+\ln\left(\frac{AR}{E_{a}g(\alpha )}\right)$$ 1

where β, α, *E*_a_, *R*, *A,* and *g*(α) are heating rate, conversion rate, apparent activation energy, ideal gas constant, apparent frequency factor, and reaction model, respectively. Amorphous TFD, annealed at −20 and 25 °C for 40 days, was collected from chambers and heated from 25 to 80 °C at a rate of 10 °C/min, held isothermally for 1 min, and then heated from 80 to 160 °C at rates of 0.5, 1, 2, and 3 °C/min in DSC. The first heating rate of 10 °C/min was introduced to minimize nucleation on heating up to 80 °C, especially during heating at the lowest rate. The reaction model of Avrami-Erofeev^39^ was used to calculate the apparent frequency factor, in which the Avrami exponent determined for the isothermal crystallization behavior at 100 °C was used. Analysis of covariance (ANCOVA) was performed to compare the values of slopes and intercepts between the samples with different annealing conditions using open-source statistical software, R.^40^ Values of *p* < 0.05 were considered significant.

### 2.4 Powder X-ray Diffraction Measurement

Amorphous TFD, annealed at −20 and 25 °C for 40 days, was collected from chambers and heated from 25 to 140 °C, which is sufficiently above the cold crystallization temperature and approximately 6 °C below *T*_m_ at a rate of 1 °C/min, held isothermally for 5 min to complete crystal growth, and then cooled to 25 °C at a rate of 20 °C/min in DSC. The lids of DSC pans were removed using forceps, and samples were carefully collected from the DSC pans using a spatula. These samples were placed between Mylar film and used for powder X-ray diffraction (PXRD) measurement on a SmartLab (Rigaku, Tokyo, Japan) equipped with a HiPix-3000 detector using Cu Kα radiation in transmission mode. The voltage and current were set to 45 kV and 200 mA, respectively. Data were collected at a scan speed of 10 °/min for a 2θ range of 2.5–40° at an interval of 0.01°. After PXRD measurement, the samples were heated from 25 to 160 °C at a rate of 1 °C/min in DSC to check the melting behaviors.

PXRD patterns were analyzed to compare the ratio of polymorphs between the samples with different annealing conditions using SmartLab Studio II (Rigaku). Peak area was calculated by profile fitting of PXRD patterns using the split pseudo-Voigt function with a background correction. Normalized peak area was obtained by the following equation:

$$\mathrm{normalized}\;\mathrm{peak}\;\mathrm{area}=\frac{\mathrm{area}\;\mathrm{of}\;\mathrm{the}\;\mathrm{specific}\;\mathrm{peak}\;\mathrm{of}\;\mathrm{form}\;I\;\mathrm{or}\;\mathrm{form}\;\mathrm{II}}{\mathrm{area}\;\mathrm{of}\;\mathrm{the}\;\mathrm{peak}\;\mathrm{at}\;2\theta =16.1^{{}^{\circ}}}\times 1000$$ 2

### 2.5 Isothermal Crystallization Study

The isothermal crystallization behavior of amorphous TFD was evaluated by quasi-isothermal modulated DSC measurements.^41^ This methodology allows quantitative evaluation of the crystallization kinetics of amorphous materials from the decrease in the reversing heat capacity with time. The sample amount for this study was increased to 10 mg, in accordance with the finding of Harada et al. that the sample amount influences the reproducibility of the measurement of reversing heat capacity.^42^ Amorphous TFD, annealed at −20, 25, and 40 °C for 7, 20, and 40 days, was collected from chambers and heated to 100 °C immediately and held isothermally for 120 min with an amplitude and period of temperature modulation of 1 °C and 120 s, respectively, in DSC. Among the advantages of this methodology, a complete time profile of the crystallization process can be obtained in a single measurement, and temperature and humidity—both of which affect physical stability—can be more rigorously controlled than with the use of stability chambers, thereby improving reproducibility. On the other hand, it is limited by the duration of experiments, which need to be completed within a realistic measurement time. Therefore, this study was conducted at 100 °C to allow sufficiently fast crystallization of amorphous TFD. Some of the samples were subsequently heated to 200 °C at a rate of 1 °C/min to check the melting behaviors without removal from the DSC instrument.

Residual ratio of the amorphous fraction, *f*(*t*), was calculated by reversing heat capacity signals according to the following equation, based on the assumption that excess heat capacity relative to that of the crystal is proportional to the amorphous fraction:^41^

$$f(t)=\frac{C_{p}(t)-C_{p}^{\mathrm{crystal}}}{C_{p}^{\mathrm{amorphous}}-C_{p}^{\mathrm{crystal}}}\times 100$$ 3

where *t*, *C*_p_(*t*), *C*_p_^crystal^, and *C*_p_^amorphous^ are time, reversing heat capacity, and specific heat capacity of crystalline and amorphous TFD, respectively. *C*_p_^amorphous^ and *C*_p_^crystal^ were calculated as the averages of the first and last 100 data points of isothermal holding at 100 °C, respectively, where heat capacity was confirmed to be constant. Isothermal crystallization curves were fit using the modified Avrami equation:^43–45^

$$f(t)=100\times 0.9^{{\left(\frac{t}{t_{10}}\right)}^{n}}$$ 4

where *t*_10_ and *n* are the time at which 10% of the sample crystallized and the Avrami exponent, which reflects the nucleation mechanism and dimension of crystal growth, respectively. *t*_10_ and the Avrami exponent were calculated to minimize the residual sum of squares of the residual ratio of amorphous fraction using the Solver add-in in Microsoft Excel.

### 2.6 Dissolution Study

Dissolution and supersaturation behaviors were evaluated using a μDISS profiler (Pion, Billerica, MA, USA) equipped with in situ UV probes with attached probe tips of 2 mm path length. A TFD acetonitrile solution was added to JP2 fluid to prepare the test medium with TFD and acetonitrile concentrations of 40 μg/mL and 1 v/v%, respectively. Standard solution containing 60 μg/mL TFD was prepared in the same manner. Each vessel was filled with 15 mL of the test medium with temperature controlled at 25 °C. Amorphous TFD in DSC pans, annealed at −20 and 25 °C for 47 days, was collected from chambers, and the pans with the samples were attached to the bottom of the probe tips using double-sided adhesive tape. The lids of the DSC pans were not used in this study. Subsequently, 40 μL of the test medium was added to samples to ensure complete wetting of the surface before insertion into the vessels. Data were collected for 48 h at 10 min intervals in the wavelength range of 200–720 nm with stirring of the medium at 150 rpm using a magnetic stirrer bar. Concentration was calculated from the area of the peak, ranging from 240 to 280 nm, in the second-derivative UV spectra. The concentration measured using in situ UV probes was corrected based on the nominal concentration of TFD in the test medium (40 μg/mL) according to the following equation:

$$\mathrm{corrected}\;\mathrm{concentration}(t)=\frac{\mathrm{nominal}\;\mathrm{concentration}\;\mathrm{of}\;\mathrm{TFD}}{\mathrm{measured}\;\mathrm{concentration}\;\mathrm{at}\;\mathrm{the}\;\mathrm{first}\;\mathrm{point}}\times \mathrm{measured}\;\mathrm{concentration}(t)$$ 5

The crystal form of precipitates appearing in the dissolution study was evaluated by PXRD. Precipitates were collected by vacuum filtration after the dissolution study. Collected precipitates were subjected to PXRD measurements without drying.

The equilibrium solubility of form I and form II of TFD crystals was determined using the shake-flask method. 5 mg of crystalline TFD powder was added to 10 mL of JP2 fluid containing 1 v/v% acetonitrile and agitated for 48 h at 25 °C. A 1 mL portion of the test medium was filtered using a syringe filter with a pore size of 0.45 μm (GL Sciences, Tokyo, Japan). The filtrate was diluted with the same volume of acetonitrile and used for the HPLC assay. HPLC measurements were performed using a 1260 Infinity LC System (Agilent Technologies, Santa Clara, CA, USA) with a YMC-Triart C18 column (4.6 mm × 75 mm, 3 μm) (YMC, Kyoto, Japan) at 40 °C. 0.1% TFA in water and 0.1% TFA in acetonitrile were pumped isocratically in the ratio of 1:1 at a total flow rate of 1 mL/min. The injection volume was 10 μL. TFD was detected with a UV detector at a wavelength of 200 nm, and the concentration was determined using a calibration curve, for which linearity was confirmed from 5 to 50 μg/mL. The crystal form of residual solids was evaluated by PXRD using the same procedure as described above to confirm that the crystal form did not change during solubility measurements (data not shown).

### 2.7 Polarized Light Microscopy

The growth behavior of TFD crystals was investigated using a BX-51 polarized light microscope (Olympus, Tokyo, Japan) equipped with a U-POT polarizer and a U-ANT analyzer. TFD crystals were melted on a thin glass using a hot plate heated at 170 °C, cooled to ambient temperature, and then annealed at −20 °C for 1 h with silica gel. A cover glass was placed on the sample after annealing to confirm the absence of birefringence before the investigation. The samples were heated from room temperature to 90 °C immediately, held isothermally for 30 min, and then heated to 100 °C using a PN121-D heat stage (MSA Factory, Tokyo, Japan) to monitor the crystal growth behavior. Growth rate was determined by measuring the evolution of the growth front of each crystal with time at each temperature.

## 3 Results and Discussion

### 3.1 Determination of Nucleation Temperature

DSC thermograms of freshly prepared amorphous TFD and those annealed at −20 °C for 1 day are shown in Figure 2. Both samples showed a glass transition at approximately 58 °C and did not crystallize when they were heated at a rate of 10 °C/min. Accordingly, TFD is classified as class III^28^ under the classification system of the crystallization tendency of pharmaceutical glass proposed by Baird et al.^29^ The heating rate was changed from 10 to 1 °C/min to investigate whether this change influenced cold crystallization. Similarly, the fresh glass did not crystallize during heating at a rate of 1 °C/min. In contrast, amorphous TFD annealed at –20 °C for 1 day showed cold crystallization and melting when heated at a rate of 1 °C/min. We hypothesized that crystal nuclei formed during annealing at −20 °C could grow only when heated at a slow rate in DSC due to the slow crystal growth of TFD.

**Figure 2:**
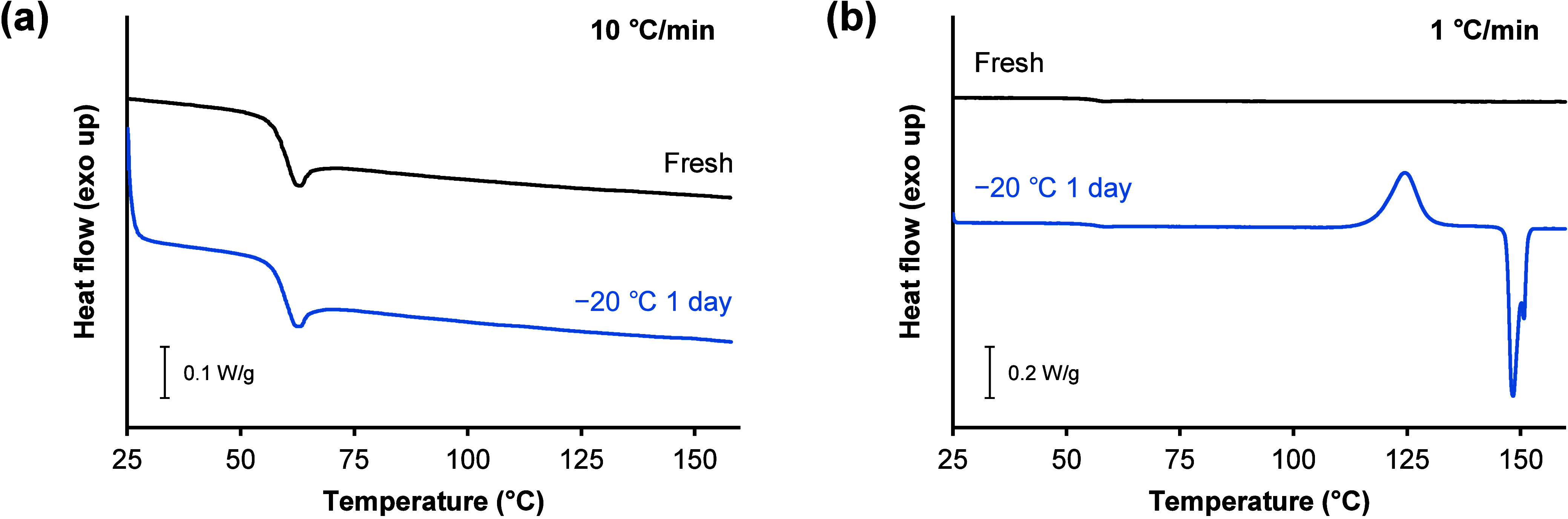
Representative DSC thermograms of freshly prepared amorphous TFD (black) and those annealed at −20 °C for 1 day (blue) with heating rates of (a) 10 and (b) 1 °C/min.

However, cold crystallization was not consistently observed, even after annealing at −20 °C, when the annealing period was short. The influence of annealing temperature and period on the probability of cold crystallization during heating at a rate of 1 °C/min in DSC is summarized in Table 2. Amorphous TFD with an annealing period shorter than 7 days showed a lower probability, particularly at higher annealing temperatures, which can be explained by the stochastic nature of nucleation and an insufficient number of crystal nuclei induced by annealing. Cold crystallization was always observed after annealing for at least 7 days at all temperatures except −80 °C. DSC thermograms of amorphous TFD annealed at various temperatures for 40 days are shown in Figure 3. A recovery peak of enthalpy relaxation was detected at *T*_g_ for samples annealed at 25 and 40 °C, with the latter showing a higher *T*_g_ induced by annealing.^46^ Amorphous TFD annealed at −30 and −20 °C showed a lower onset temperature of cold crystallization (*T*_onset_) than those annealed at higher temperatures. Two overlapping melting peaks were observed at approximately 146 and 150 °C, which are consistent with the reported *T*_m_ values of form II and form I of TFD crystals, respectively.^21,24,25^ Amorphous TFD annealed at −30 and −20 °C showed larger and smaller melting peaks of form II and form I, respectively, than those annealed at higher temperatures. The effect of annealing temperature and period on *T*_onset_ and the ratio of enthalpy of fusion of form II to form I (Δ*H*_f_II_/Δ*H*_f_I_) are shown in Figure 4. Amorphous TFD annealed at −30 and −20 °C showed lower *T*_onset_ and higher Δ*H*_f_II_/Δ*H*_f_I_ than those annealed at higher temperatures, and these trends became more pronounced with the increasing annealing period. The ratio of enthalpy of cold crystallization, Δ*H*_c_, to Δ*H*_f_ was almost constant across all samples irrespective of annealing conditions (Figure S1), indicating that crystallization did not occur during annealing.^47^ These results indicate that nucleation of form II was induced by annealing at −30 and −20 °C.

**Figure 3:**
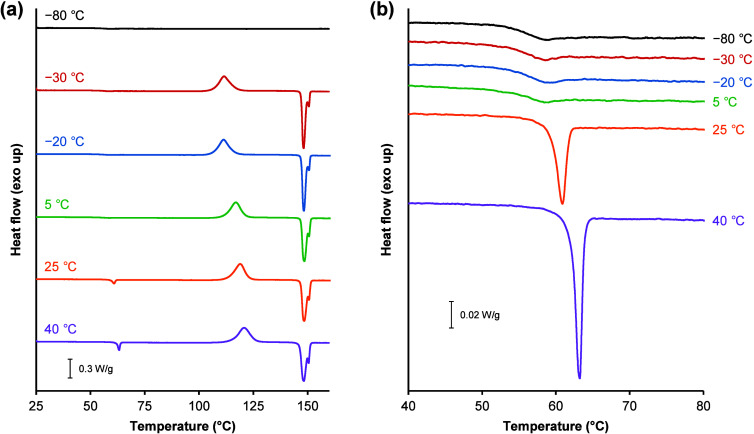
Representative DSC thermograms of amorphous TFD annealed at −80 (black), −30 (red), −20 (blue), 5 (green), 25 (orange), and 40 °C (purple) for 40 days. (a) Full scale thermograms. (b) Expanded thermograms near *T*_g_.

**Figure 4:**
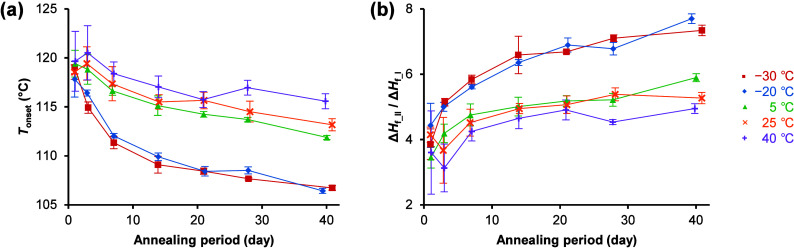
Effect of annealing temperature and period on (a) *T*_onset_ and (b) Δ*H*_f_II_/Δ*H*_f_I_ of amorphous TFD. Data are the averages of at least three experiments. Error bars indicate ± standard deviation.

**Table 2:** Effect of Annealing Temperature and Period on Probability of Cold Crystallization of Amorphous TFD^a^

	annealing temperature (°C)					
annealing period (day)	–80	–30	–20	5	25	40
1	NT	3/4	4/5	3/6	4/8	3/8
3	NT	4/4	3/3	3/4	3/4	3/4
7	NT	3/3	3/3	3/3	3/3	3/3
14	0/5	3/3	3/3	3/3	3/3	3/3
28	0/5	3/3	3/3	3/3	3/3	3/3
40	1/5	3/3	3/3	3/3	3/3	3/3

a NT: not tested. The numerator and denominator of the fractions represent the number of samples that showed cold crystallization and tested samples, respectively.

### 3.2 PXRD Measurement of TFD Crystallized from the Amorphous Form

PXRD measurements were performed to evaluate the crystal form of TFD after cold crystallization from the amorphous form. Amorphous TFD annealed at −20 and 25 °C for 40 days was crystallized by heating to just below *T*_m_ in DSC and collected for PXRD measurements. The crystal form of both samples was predominantly form II; however, at the same time, characteristic peaks of form I were weakly detected (Figures 5 and S2). To compare the ratio of polymorphs between the samples with different annealing conditions, the areas of the specific peaks of form I and form II were normalized by the area of the peak at 2θ = 16.1° (Table 3). Amorphous TFD annealed at −20 °C contained a slightly larger amount of form II crystals than that annealed at 25 °C. Δ*H*_f_II_/Δ*H*_f_I_ values of samples used for the PXRD measurements were 7.8 (−20 °C) and 4.7 (25 °C) (Figure S3), which are consistent with the results shown in Figure 4b.

**Figure 5:**
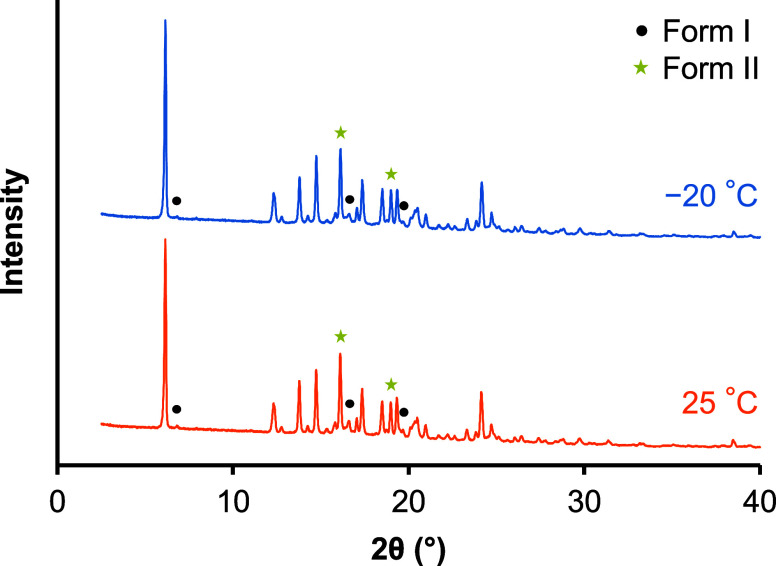
PXRD patterns of TFD crystallized by heating to just below *T*_m_. Samples were annealed at −20 (blue) and 25 °C (orange) for 40 days before the measurement. Specific peaks of form I (black circle) and form II (yellow star) are indicated in the figure.

**Table 3:** Normalized Area of Specific Peaks of Form I and Form II of TFD Crystals in PXRD Patterns^a^

	normalized peak area^b^		
2θ (°)	–20 °C 40 days	25 °C 40 days	peak origin
6.8	43	53	form I
16.1	1000	1000	form II
16.6	267	287	form I
19.0	437	396	form II
19.7	64	156	form I

a Annealed amorphous TFD was crystallized by heating to just below *T*_m_ before the PXRD measurement.

b Peak areas were normalized with the area of the peak at 2θ = 16.1° as 1000.

### 3.3 Isothermal Crystallization Behavior

The effect of annealing temperature and period on subsequent isothermal crystallization behavior of amorphous TFD was evaluated by quasi-isothermal modulated DSC.^41^ DSC thermograms of amorphous TFD annealed at −20, 25, and 40 °C for 7, 20, and 40 days during isothermal holding at 100 °C are shown in Figure S4. Heat capacity was converted to the residual ratio of amorphous fraction using eq 3 and fit with the modified Avrami equation^45^ (eq 4) (Figure 6). Amorphous TFD annealed at −20 °C showed the fastest depression in the curve, followed by those annealed at 25 and 40 °C, regardless of the annealing period. Values of *t*_10_ and the Avrami exponent are shown in Table 4. The *t*_10_ of samples annealed at −20 °C was smaller than that of samples annealed at 25 and 40 °C, indicating that physical stability was worsened in the presence of preformed crystal nuclei. The Avrami exponent of samples annealed at −20 °C was calculated to be approximately 5. The Avrami exponent indicates the mechanisms of nucleation and crystal growth and generally takes values from 1 to 4. The value larger than 4 may be interpreted by a time-dependent increase in nucleation rate.^48,49^ Amorphous TFD was confirmed to crystallize in form II during isothermal holding at 100 °C based on melting behavior (Figure S5). However, closer inspection of the thermogram of amorphous TFD annealed at −20 °C reveals a small shoulder peak of melting of form I crystals, which would be evidence of cross nucleation of form I, discussed later, and might affect the Avrami exponent.

**Figure 6:**
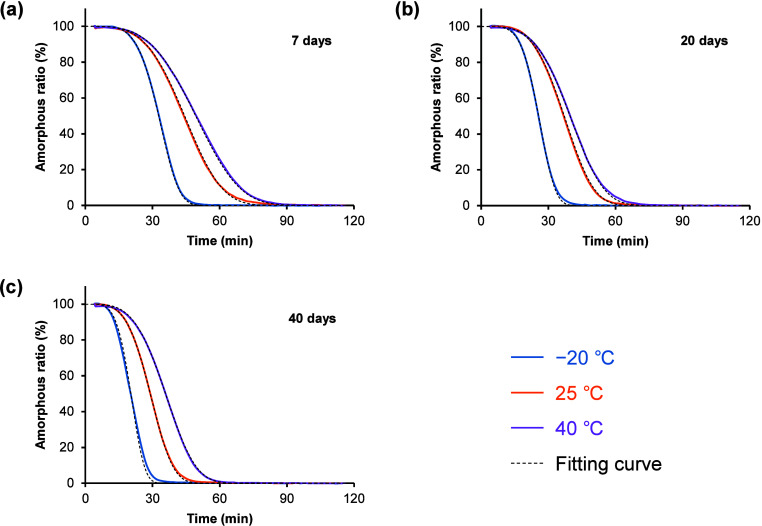
Isothermal crystallization behavior of amorphous TFD at 100 °C. Samples were preannealed at −20 (blue), 25 (orange), and 40 °C (purple) for (a) 7, (b) 20, and (c) 40 days before the evaluations. Data are the average of at least four experiments without error bars for clarity. All curves are fit by the modified Avrami equation (dotted line).

**Table 4:** Values of *t*_10_ and the Avrami Exponent Calculated by the Modified Avrami Equation^a^

annealing temperature (°C)	annealing period (day)	*t*_10_(min)	Avrami exponent
–20		22.6 ± 1.2	5.1 ± 0.4
25	7	27.0 ± 1.2	3.8 ± 0.2
40		29.2 ± 1.5	3.6 ± 0.1
–20		17.5 ± 0.9	4.9 ± 0.2
25	20	23.2 ± 0.7	4.0 ± 0.1
40		24.2 ± 1.1	3.8 ± 0.3
–20		13.6 ± 2.9	4.7 ± 0.9
25	40	18.3 ± 0.7	4.1 ± 0.3
40		21.8 ± 0.2	3.8 ± 0.1

a *t*_10_: The time at which 10% of the sample crystallized. *n* = 4–5 for each condition. Data are mean ± standard deviation.

Previous research on the effect of nucleation on the physical stability of celecoxib glass revealed that nucleated glass showed slightly faster crystallization than fresh glass at 35 °C, whereas the effect of nucleation was not obvious at temperatures higher than 40 °C due to the simultaneous crystallization of a different form.^17^ This finding highlights the importance of the resultant crystal form when the effect of nucleation on the physical stability is investigated. Crystallization experiments using many individual samples often pose problems of reproducibility.^17,45,50^ We were able to obtain complete profiles of the isothermal crystallization behavior of amorphous TFD with high reproducibility using quasi-isothermal modulated DSC.

### 3.4 Dissolution Study

The effect of the annealing temperature on the dissolution and supersaturation behaviors of amorphous TFD was evaluated using a μDISS Profiler. TFD was predissolved in JP2 fluid at a concentration of 40 μg/mL to increase the degree of supersaturation and enhance precipitation because the dissolution rate of amorphous TFD prepared in DSC pans was markedly slow due to the small surface area available for dissolution. Dissolution and supersaturation behaviors of amorphous TFD annealed at −20 and 25 °C for 47 days were closely similar, wherein the concentration of TFD reached a maximum at around 24 h and then started to decrease due to precipitation (Figure 7). The final concentration in the dissolution study was higher than the equilibrium solubility of form I (13.6 ± 0.1 μg/mL) and form II (14.3 ± 0.3 μg/mL) of TFD crystals. PXRD patterns of the precipitates were similar under both annealing conditions but differed from that of form II (Figure 8). The crystallinity of the precipitate decreased after the precipitate was vacuum-dried at 50 °C overnight (Figure S6). In the thermogravimetric analysis and DSC measurement of the precipitate, weight loss and a broad endothermic peak, respectively, were observed below 100 °C (Figure S7). Furthermore, a large weight loss was observed below 20% relative humidity (RH) under dynamic vapor sorption analysis of the precipitate (Figure S8), indicating the presence of water molecules incorporated in the crystal lattice as channel water.^51^ Taken together, the precipitate was considered to be a hydrate of TFD crystals based on these characterizations.

**Figure 7:**
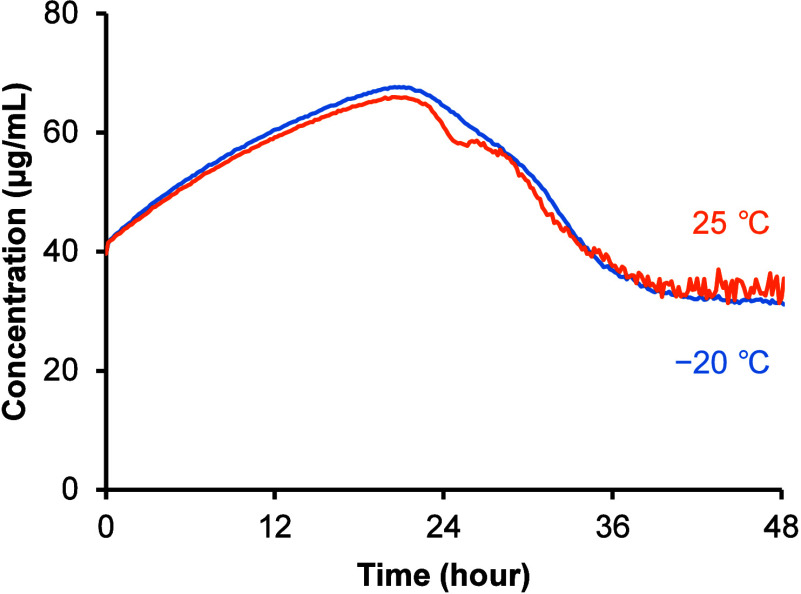
Dissolution behavior of amorphous TFD annealed at −20 (blue) and 25 °C (orange) for 47 days. The test medium was JP2 fluid containing 40 μg/mL TFD. Data are the average of six experiments without error bars for clarity. The equilibrium solubility of form I and form II were 13.6 ± 0.1 and 14.3 ± 0.3 μg/mL, respectively.

**Figure 8:**
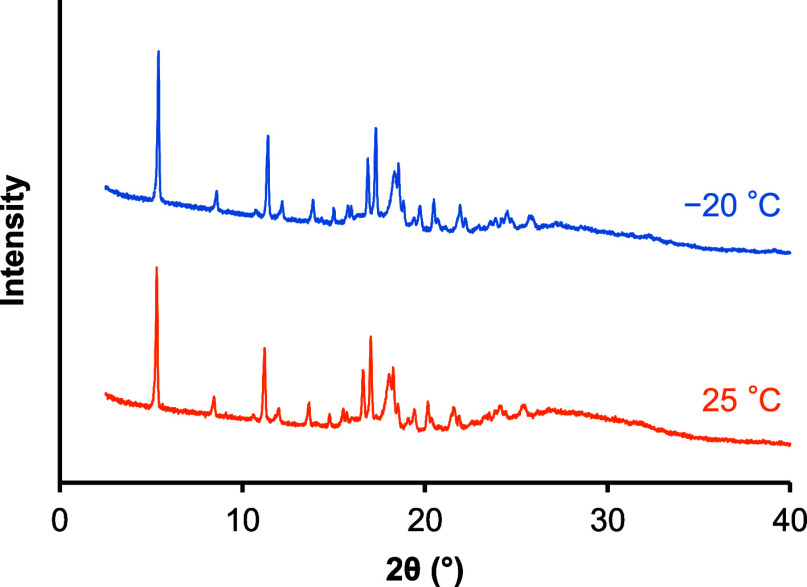
PXRD patterns of precipitates obtained from the dissolution study of amorphous TFD annealed at −20 (blue) and 25 °C (orange) for 47 days.

Yao et al. and our group previously reported that the dissolution and supersaturation performances of some amorphous compounds deteriorated in the presence of crystal nuclei.^18,52^ In these studies, crystal nuclei worked as templates for crystallization, and the resultant crystal form was consistent with that of nuclei, except in the case of ritonavir, where form III nuclei induced heterogeneous crystallization of form I.^52^ These findings indicate that crystal nuclei can directly and even indirectly enhance precipitation. Although amorphous TFD annealed at −20 °C contained a larger amount of form II nuclei than that annealed at 25 °C, as revealed by DSC measurements, the dissolution and supersaturation behaviors of these samples were closely similar. Therefore, nucleation and crystal growth of TFD hydrate would have occurred during the dissolution study without being influenced by the form II nuclei.

### 3.5 Isoconversional Analysis of Cold Crystallization Kinetics

Heating rate dependence of the cold crystallization behavior was evaluated to elucidate the effect of nucleation on crystal growth kinetics. DSC thermograms of amorphous TFD annealed at −20 and 25 °C for 40 days with various heating rates are shown in Figure S9, and thermodynamic parameters of cold crystallization and melting are summarized in Table 5. *T*_onset_ was strongly affected by heating rate, and crystal growth was not completed even at a heating rate of 3 °C/min, indicating a slow crystal growth rate of TFD. The KAS analysis was performed to quantitatively evaluate the effect of nucleation on crystal growth kinetics using the data obtained at heating rates of 0.5, 1, and 2 °C/min (Figures 9 and S10). The apparent activation energy monotonically decreased with increasing α, indicating that the process has multistep kinetics.^39,53^ Since the KAS equation is derived based on the assumption of single-step kinetics, a systematic error in the apparent activation energy becomes large when it changes with α.^39^ Thus, focus was made on the small conversion range, α ≤ 10%, where preformed crystal nuclei strongly affect the kinetics of cold crystallization, to calculate the apparent frequency factor with the Avrami–Erofeev model (Table 6). The difference in the apparent activation energy between the samples with different annealing conditions was not significant. In contrast, the intercept for amorphous TFD annealed at −20 °C was significantly larger than that of samples annealed at 25 °C, which is probably attributed to the difference in the apparent frequency factor, as the apparent activation energy did not significantly differ. The frequency factor typically reflects the frequency of successful molecular collisions leading to transformation. Therefore, the larger apparent frequency factor for samples annealed at −20 °C probably reflects a larger amount of preformed crystal nuclei.

**Figure 9:**
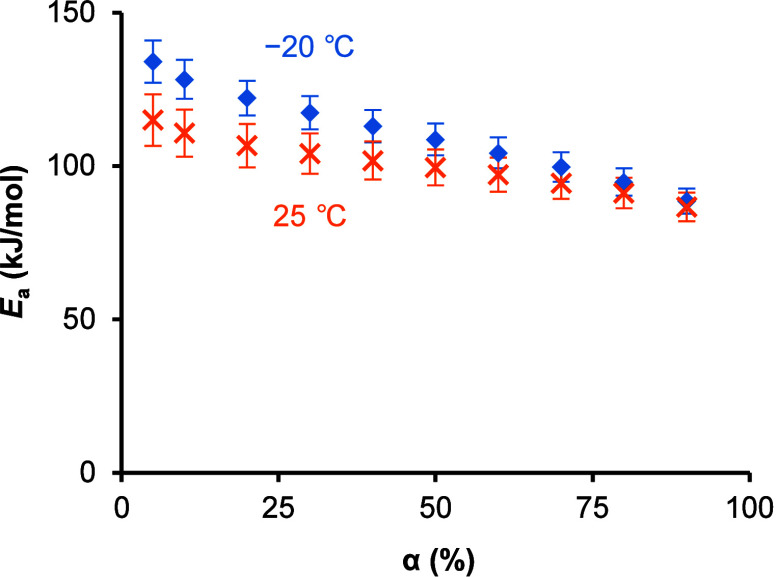
Dependence of the apparent activation energy of cold crystallization on conversion rate of amorphous TFD annealed at −20 (blue diamond) and 25 °C (orange cross) for 40 days. Error bars indicate ± standard error.

**Table 5:** Heating Rate Dependence of Cold Crystallization and Melting Behaviors of TFD^a^

annealing condition	heating rate (°C/min)	*T*_onset_ (°C)	Δ*H*_c_ (J/g)	Δ*H*_f_ (J/g)	Δ*H*_f_II_/Δ*H*_f_I_
–20 °C 40 days	0.5	102.6 ± 0.1	92.6 ± 0.9	114.7 ± 1.5	15.4 ± 0.1
	1	107.4 ± 0.4	95.3 ± 1.5	113.2 ± 1.2	7.4 ± 0.2
	2	113.9 ± 0.3	100.0 ± 1.2	112.0 ± 1.5	4.5 ± 0.1
	3	118.5 ± 0.3	NA	78.4 ± 2.7	3.4 ± 0.1
25 °C 40 days	0.5	105.9 ± 2.1	95.0 ± 1.4	113.2 ± 0.1	10.1 ± 3.6
	1	113.7 ± 1.4	98.5 ± 0.2	112.9 ± 0.2	5.4 ± 0.3
	2	120.4 ± 0.8	89.6 ± 3.2	99.3 ± 4.9	3.3 ± 0.2
	3	123.1 ± 0.9	NA	39.2 ± 8.8	2.4 ± 0.2

a Δ*H*_f_ is the sum of Δ*H*_f_I_ and Δ*H*_f_II_. NA: not available due to incomplete crystallization. *n* = 3 for each condition. Data are mean ± standard deviation.

**Table 6:** Apparent Activation Energy and Apparent Frequency Factor of Cold Crystallization Process in Amorphous TFD Annealed at −20 and 25 °C for 40 Days

α (%)	annealing condition	*E*_a_ (kJ/mol)	$\ln\left(\frac{AR}{E_{a}g\left(\alpha \right)}\right)$	ln *A*	*R* ^2^
5	–20 °C 40 days	134.0 ± 6.9	54.2 ± 2.2	63.3 ± 2.2	0.982
	25 °C 40 days	115.0 ± 8.4	47.7 ± 2.6	56.5 ± 2.6	0.964
	*p*-value^a^	0.11	<0.0001	NA	NA
10	–20 °C 40 days	128.2 ± 6.3	52.2 ± 2.0	61.4 ± 2.0	0.983
	25 °C 40 days	110.7 ± 7.7	46.3 ± 2.4	55.2 ± 2.4	0.967
	*p*-value^a^	0.11	<0.0001	NA	NA

a Calculated by ANCOVA. *R* ^2^: coefficient of determination. NA: not available. Error limits indicate ± standard error.

Δ*H*_f_II_/Δ*H*_f_I_ of amorphous TFD annealed at −20 and 25 °C for 40 days decreased with an increase in heating rate (Table 5), even though the amount of preformed crystal nuclei present initially is the same irrespective of heating rate. Δ*H*_f_II_/Δ*H*_f_I_ and *T*_onset_ showed a linear correlation regardless of annealing conditions and heating rates when Δ*H*_f_II_/Δ*H*_f_I_ was at least less than 10 (Figure S11). These results indicate that crystallization to form II and form I preferentially occurred at lower and higher temperatures, respectively. This observation is consistent with a previous study by Yonemochi et al., in which amorphous TFD crystallized at 135 °C with a higher percentage of form I content than when crystallized at 120 °C.^24^ Taken together, the increase in heating rate deprived form II of time for crystal growth and increased the crystal growth of form I at a higher temperature, thereby resulting in a lower Δ*H*_f_II_/Δ*H*_f_I_.

### 3.6 Mechanism of Nucleation of Form I

The crystal form of nuclei cannot be directly identified by any analytical method due to their extremely small size.^54–56^ In this study, the crystal nuclei in amorphous TFD induced by annealing were indirectly determined to be those for form II from the results of DSC experiments, while a question remained about when and how the nucleation of form I occurred. Form I is more stable than form II with a thermodynamically monotropic relationship, and the nucleation of form II is therefore probably kinetically more favorable than form I according to Ostwald’s rule.^57,58^ Solid-state transformation of form II to form I during heating in DSC is not likely since a slower heating rate led to a higher Δ*H*_f_II_/Δ*H*_f_I_ (Table 5). Accordingly, annealing should have directly or indirectly induced nucleation of form I because cold crystallization was not observed for the fresh glass. The growth behaviors of these two polymorphs were investigated using PLM (Figures 10, S12, S13, Movies S1, and S2). Crystal growth of form II was characterized by linear extension of needles with irregular edges at 90 °C. Form I crystals with prism-like morphology then started to grow uniformly with a smooth growth front from the form II crystals when the temperature was increased to 100 °C. Form II crystals predominantly grew from the preformed form II nuclei in spite of essentially the same growth rate, 0.02 ± 0.01 μm/s and 0.02 ± 0.00 μm/s for form I and form II, respectively, at 90 °C. Meanwhile, crystallization of form I was enhanced with an increase in the growth rate (0.11 ± 0.02 μm/s) and the presence of grown form II crystals at 100 °C. Crystallization to form I appeared to be induced by cross nucleation^8,59^ from these observations, i.e., form II nuclei or crystals assisted the nucleation of form I. Furthermore, these results are consistent with the relationship between *T*_onset_ and Δ*H*_f_II_/Δ*H*_f_I_ discussed in the previous section and indicate that nucleation of form I probably occurred during heating in DSC measurements. In the systematic investigation by Trasi et al., the crystal growth rate of pharmaceutical compounds ranges from ca. 0.02 to 216 μm/s.^60^ The slow growth rate of TFD crystals explains why slow heating is required to observe cold crystallization in the DSC measurement.

**Figure 10:**
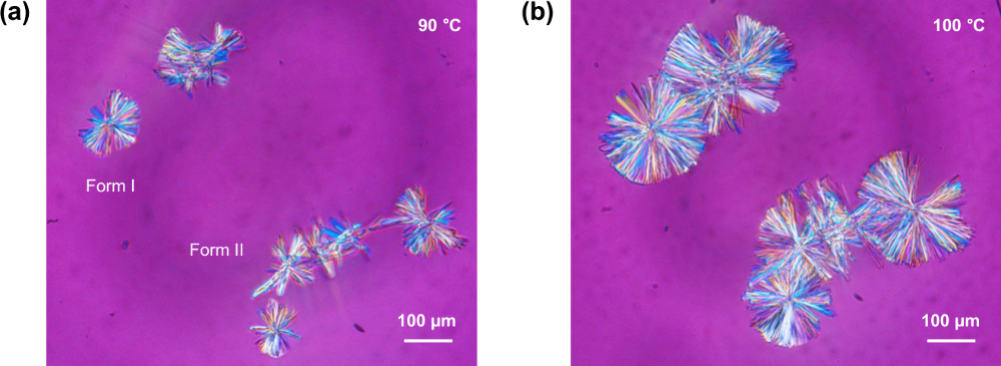
PLM images of amorphous TFD heated at (a) 90 and (b) 100 °C. Samples were preannealed at −20 °C for 1 h before the investigation.

### 3.7 Impact of Crack Formation on Nucleation

Amorphization by quench cooling of a melt sometimes leads to crack formation in a sample, which is believed to enhance crystallization.^12,16,61,62^ Sharp spikes were observed below 20 °C during quench cooling of the TFD melt in DSC (Figure S14). These peaks are indicative of crack formation in samples, which can be caused by the mechanical stress in the glass during a rapid change in the volume.^16,63^ These peaks were seldom observed above 20 °C during quench cooling of the TFD melt, therefore indicating that amorphous TFD had no cracks before annealing. However, cracks were observed in amorphous TFD after annealing at −20 °C for 7 days, whereas those annealed at higher temperatures showed no cracks (Figure S15). In order to investigate the impact of crack formation on the nucleation of form II, an additional annealing step at −20 °C for 10 min was introduced to intentionally induce the cracks before annealing at target temperatures. All samples showed sharp peaks indicative of crack formation during quench cooling of the melt in DSC, and the cracks were maintained after annealing at target temperatures for 7 days (Figure S15). The impact of crack formation on *T*_onset_ of amorphous TFD annealed at target temperatures for 7 days is shown in Figure 11. *T*_onset_ of amorphous TFD annealed at 5 °C decreased to a similar level to that annealed at −20 °C, while those annealed at 25 and 40 °C were not affected by cracks. These results indicate that the nucleation temperature of amorphous TFD is indeed much lower than *T*_g_ although crack formation might partially influence the nucleation behavior. Moreover, the fact that amorphous TFD annealed at −80 °C seldom showed cold crystallization during heating in DSC (Table 2) also supports the idea that our results cannot be explained by crack formation alone.

**Figure 11:**
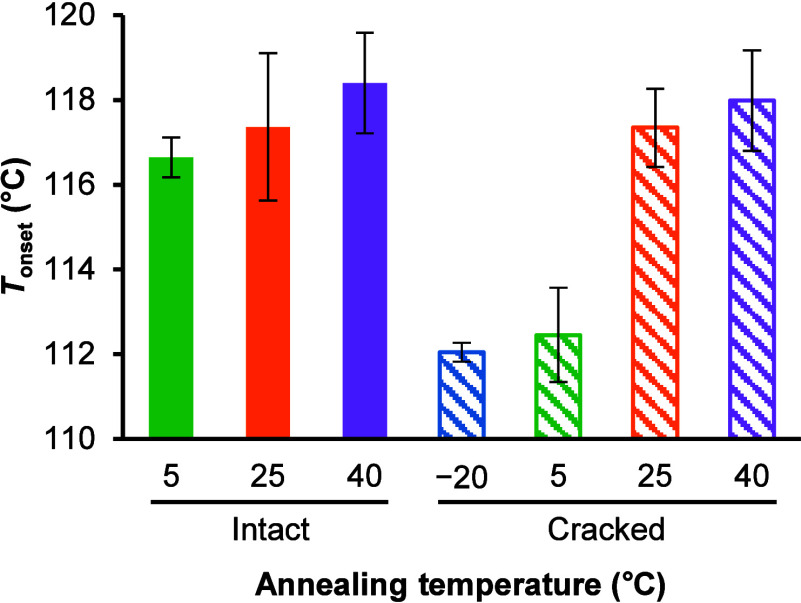
Impact of crack formation on *T*_onset_ of amorphous TFD annealed at various temperatures for 7 days. Data are the average of at least three experiments. Error bars indicate ± standard deviation.

### 3.8 Role of Local Molecular Mobility in the Nucleation Process below *T*_g_

Nucleation rate is determined by the balance of thermodynamic driving force, interfacial energy between crystal nuclei and surrounding molecules, and molecular mobility.^9,64^ A maximum nucleation rate is generally found just above *T*_g_, where global molecular mobility facilitates structural rearrangements.^8^ Dudognon et al. and Bama et al. evaluated the molecular mobility of amorphous TFD using dielectric relaxation spectroscopy.^20,31^ α relaxation was observed above the *T*_g_. The temperature of zero mobility, *T*_0_, calculated from the Vogel–Tammann–Fulcher–Hesse equation was 10 °C,^20^ indicating that α relaxation is not active in freezing temperatures. On the other hand, γ relaxation was observed in the temperature range of 173 K to ambient temperature, which was linked to the molecular motion of a large part of the molecule derived from the flexible central alkyl chain by molecular dynamics simulations.^20^ Meanwhile, β relaxation was not observed due to a very weak magnitude and the proximity of the γ relaxation.^20^ Furthermore, supplementary relaxation, which is observable only in the presence of a few percent of water molecules, was detected at a lower frequency in the same temperature range as the γ relaxation.^31^ Amorphous TFD annealed at −20 and 25 °C for 7 days showed no weight loss during thermogravimetric analysis (Figure S16), indicating that the supplementary process probably did not occur in this study. Taken together, γ relaxation seems to act as a facilitator of nucleation in amorphous TFD at around −20 °C. The fact that nucleation was not induced in amorphous TFD by annealing at −80 °C can be explained by the slower γ process at that temperature.

According to classical nucleation theory, the fate of crystal nuclei is determined by their size.^64^ Once the radius of crystal nuclei exceeds the critical value, r*, nuclei can continue to grow.

$$r^{*}=\frac{2\sigma T_{m}}{\Delta H_{f}(T_{m}-T)}$$ 6

where σ is the specific surface energy of the interface between crystal nuclei and surrounding molecules. Since σ is nearly constant or decreases with decreasing temperature,^65–67^ the critical radius for nucleation decreases with decreasing temperature even below *T*_g_. Therefore, although global molecular mobility was frozen in the optimum nucleation temperature range for form II, the nucleation could be facilitated by the γ process, which enables association of molecules for nucleation without molecular rearrangement, possibly assisted by a reduction in the critical radius for nucleation. Accordingly, the nucleation at temperatures much lower than *T*_g_ can be understood by the interplay of thermodynamic driving force, interfacial energy, and molecular mobility, in the same way as the nucleation occurring near *T*_g_.

## 4 Conclusions

The crystallization behavior of amorphous TFD was investigated by focusing on the nucleation temperature. The nucleation rate of form II was enhanced after annealing at a temperature much lower than *T*_g_, which was revealed by observation of cold crystallization behavior after annealing. The effect of nucleation on the physical stability of amorphous TFD was evaluated by two approaches. The isothermal crystallization rate of amorphous TFD was faster for those with preformed crystal nuclei. Meanwhile, the dissolution and supersaturation behaviors were not affected by nucleation since form II nuclei did not work as templates for the crystal form appearing in the supersaturated solution. Preformed crystal nuclei induced by annealing likely increased the apparent frequency factor of the cold crystallization process, as demonstrated by the KAS analysis. The competition in crystal growth between form I and form II, wherein form II predominantly grew over form I at a lower temperature, was revealed by PLM and the correlation between *T*_onset_ and Δ*H*_f_II_/Δ*H*_f_I_. Although crack formation partially affected this nucleation behavior, it was not the dominant factor. These observations emphasize the importance of understanding the nucleation behavior of amorphous solids and its impact on physical stability. Although storage at low temperatures is generally favored with the expectation that it will minimize or prevent physical and chemical changes in pharmaceutical formulations, this may not be true for amorphous dosage forms.

## Supplementary Material







## Data Availability

The data underlying this study are available from the corresponding author upon request.

## Notes

Relationship between Δ*H*_c_ and Δ*H*_f_ (Figure S1); PXRD patterns of form I and form II (Figure S2); DSC thermograms of TFD after PXRD measurement (Figure S3); DSC thermograms of amorphous TFD during isothermal crystallization study (Figure S4); DSC thermograms of TFD after isothermal crystallization study (Figure S5); PXRD patterns of the precipitate (Figure S6); thermal analysis of the precipitate (Figure S7); dynamic vapor sorption analysis of the precipitate (Figure S8); DSC thermograms of amorphous TFD with various heating rates (Figure S9); KAS plots for cold crystallization process (Figure S10); relationship between *T*_onset_ and Δ*H*_f_II_/Δ*H*_f_I_ (Figure S11); PLM images of TFD crystals (Figure S12); temperature dependence of growth rate of form I (Figure S13); DSC thermograms of TFD melt (Figure S14); microscopic images of amorphous TFD in DSC pans (Figure S15); and thermogravimetric analysis of amorphous TFD (Figure S16) (PDF)
Growth behavior of TFD crystals at 90 °C (MP4)
Growth behavior of TFD crystals at 100 °C (MP4)
