# Supplementary material for: Nucleation in Amorphous Terfenadine at a Temperature Much Lower than the Glass Transition Temperature and Its Impact on Physical Stability
Source: Mol Pharm. 2025 Aug 25;22(10):6038–49. doi: 10.1021/acs.molpharmaceut.5c00700 (PMC12505274; doi:10.1021/acs.molpharmaceut.5c00700)
Supplement: Supplementary file 1 [file mp5c00700_si_001.pdf]

Supporting Information for

Nucleation in amorphous terfenadine at a temperature much lower than the glass transition temperature and its impact on physical stability

Katsutoshi Yamaguchi [1], Yuya Ishizuka [2], Etsushi Yoshikawa [2], Takashi Makishima [2], Ryo Mizoguchi [1] and Kohsaku Kawakami [3, 4]

[1] Pharmaceutical Developability, CMC Research, Astellas Pharma Inc., 21 Miyukigaoka, Tsukuba, Ibaraki 3058585, Japan

[2] Analytical Research 1, Analytical Research Labs., CMC Development, Astellas Pharma Inc., 21 Miyukigaoka, Tsukuba, Ibaraki 3058585, Japan

[3] Medical Soft Matter Group, Research Center for Macromolecules and Biomaterials, National Institute for Materials Science, 1-1 Namiki, Tsukuba, Ibaraki 3050044, Japan

[4] Graduate School of Science and Technology, University of Tsukuba, 1-1-1 Tennodai, Tsukuba, Ibaraki 3058577, Japan

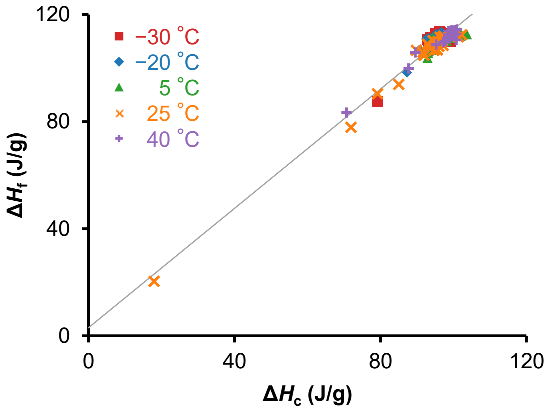

Figure S1. Relationship between ΔH_c and ΔH_f of amorphous TFD annealed at −30 °C (red square), −20 °C (blue diamond), 5 °C (green triangle), 25 °C (orange cross) and 40 °C (purple plus). ΔH_f is the sum of ΔH_{f_I} and ΔH_{f_II}.

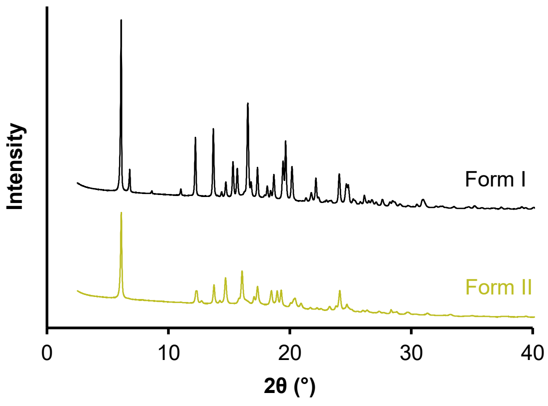

Figure S2. PXRD patterns of form I (black) and form II (yellow) of TFD crystals.

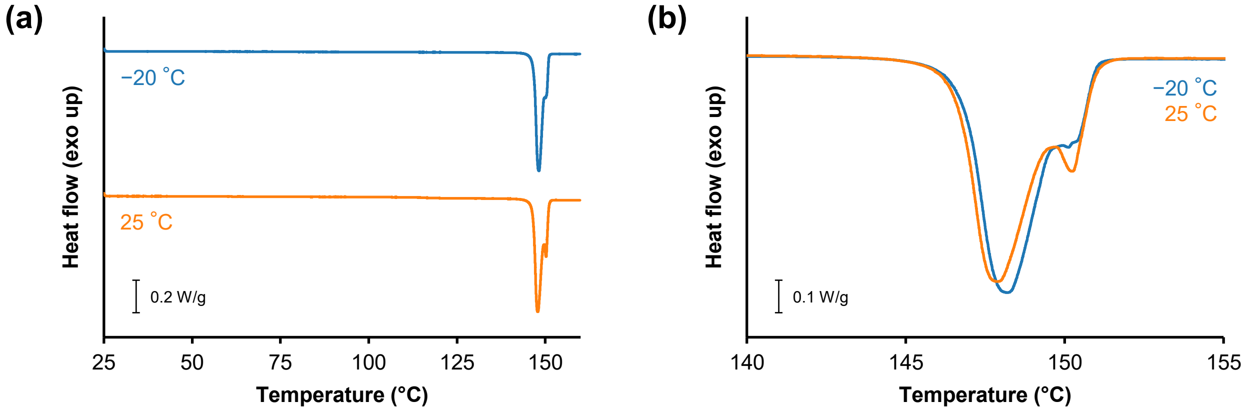

Figure S3. DSC thermograms of TFD after PXRD measurement. Amorphous TFD annealed at −20 °C (blue) and 25 °C (orange) for 40 days were crystallized by heating to just below T_m and used for the measurements. (a) Full scale thermograms. (b) Expanded thermograms near T_m.

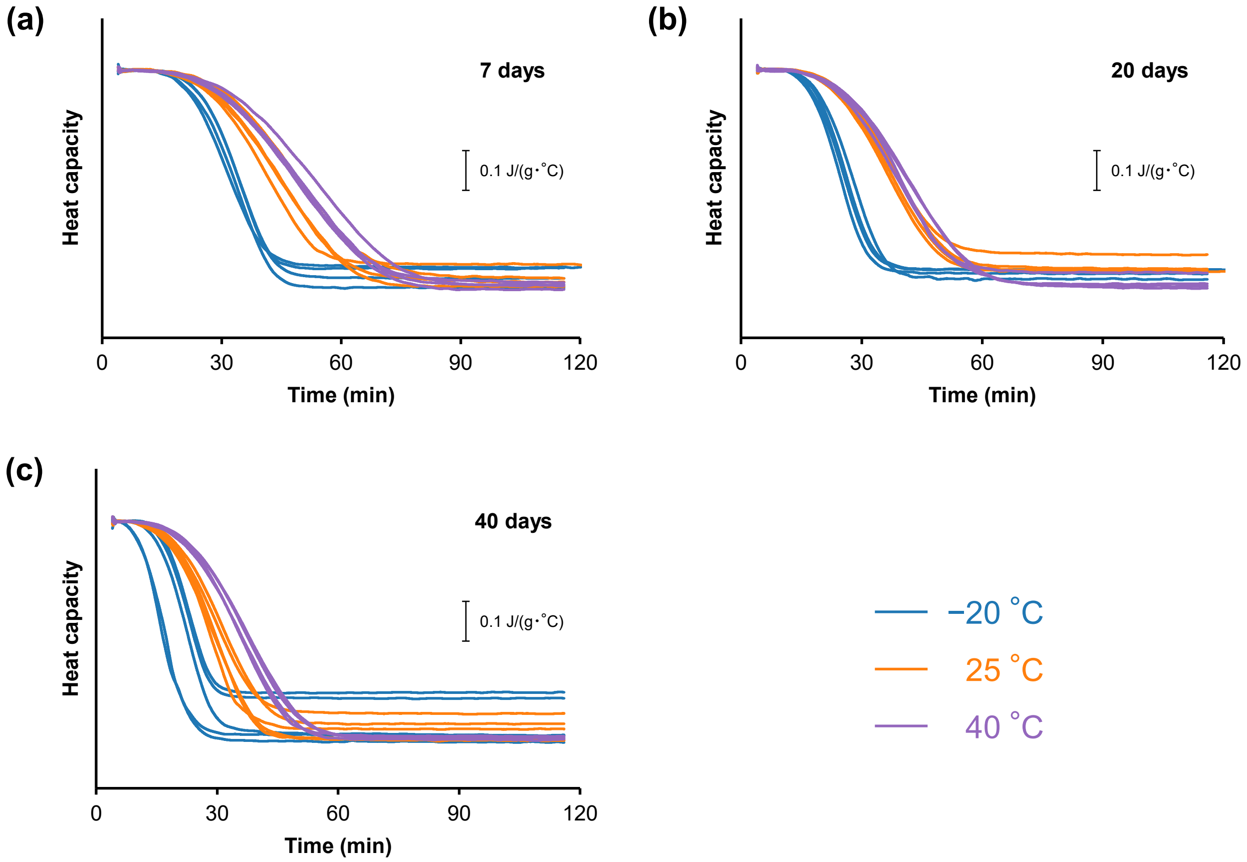

Figure S4. DSC thermograms of amorphous TFD with quasi-isothermal temperature modulation at 100 °C. Samples were pre-annealed at −20 °C (blue), 25 °C (orange) and 40 °C (purple) for (a) 7 days, (b) 20 days and (c) 40 days before the measurements.

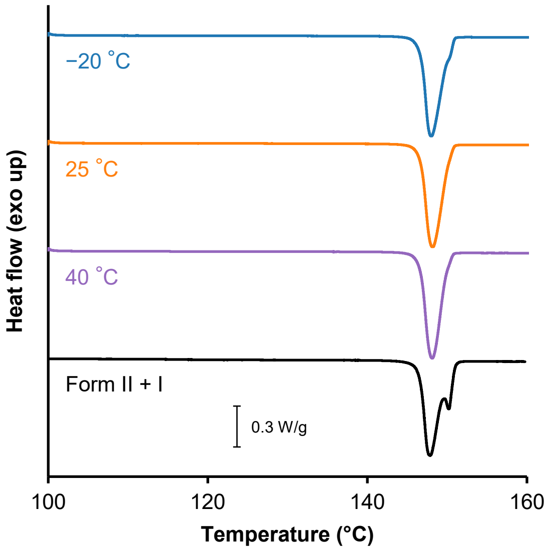

Figure S5. Representative DSC thermograms of TFD after isothermal crystallization at 100 °C. Amorphous TFD annealed at −20 °C (blue), 25 °C (orange) and 40 °C (purple) for 40 days were crystallized by isothermal holding at 100 °C and subjected to measurement. A thermogram of form II of TFD crystals containing a small amount of form I (black), which is also shown in Figure S3, is shown at the bottom for comparison.

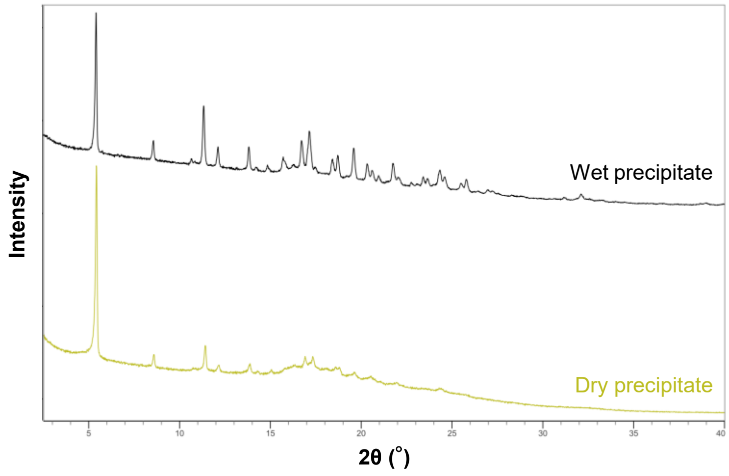

Figure S6. PXRD patterns of the precipitate obtained in the dissolution study before and after drying. Dry samples were prepared by vacuum drying the wet samples at 50 °C overnight.

Thermal analysis of the precipitate obtained in the dissolution study

DSC measurement of the precipitate was performed using a DSC 2500 (TA Instruments) under a nitrogen atmosphere with a flow rate of 50 mL/min. 3 mg of the precipitate was weighed in a Tzero pan (TA Instruments) and heated from 25 °C to 160 °C at a rate of 10 °C/min.

Thermogravimetric analysis (TGA) of the precipitate was performed using a TGA 5500 (TA Instruments) under a nitrogen atmosphere with a flow rate of 25 mL/min. 5 mg of the precipitate was weighed in a Tzero pan and placed on a platinum sample pan for TGA (TA Instruments). Samples were heated from 25 °C to 200 °C at a rate of 10 °C/min.

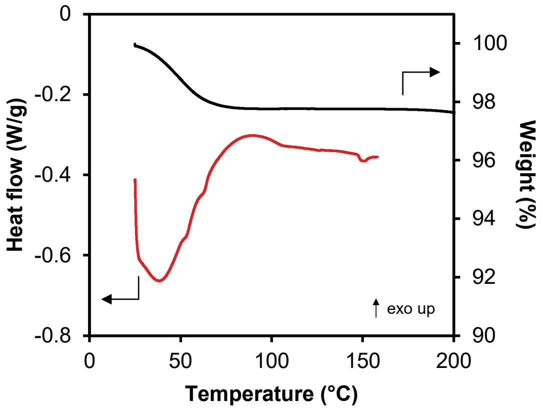

Figure S7. DSC (red) and TGA (black) thermograms of the precipitate obtained in the dissolution study. The weight loss (%) was 2.2, which corresponds to 0.6 equivalent moles of water molecules.

Dynamic vapor sorption analysis of the precipitate obtained in the dissolution study

Dynamic vapor sorption analysis of the precipitate obtained in the dissolution study was performed at 25 °C using a Q5000SA water sorption analyzer (TA Instruments). Approximately 5 mg of the precipitate was loaded onto the sample pan and initially equilibrated at 60% RH for 180 min. Then, the relative humidity was changed from 60 → 95 → 5 → 60% RH with increments of 5% RH. The equilibrium condition was set to <0.03% of weight change over 10 min or maximum dwell time of 180 min at each step.

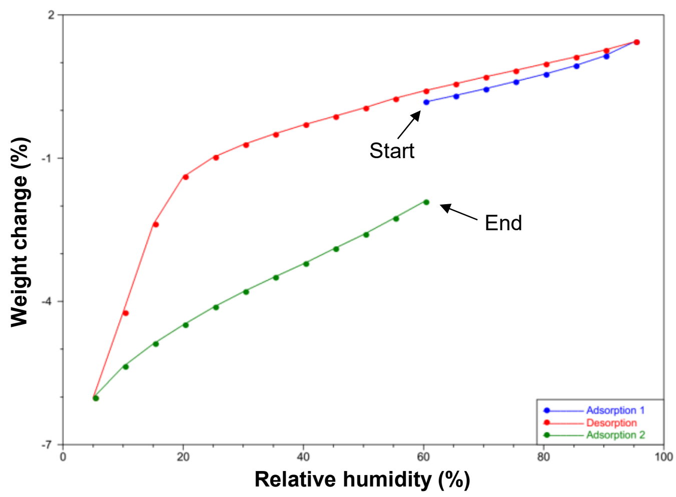

Figure S8. Dynamic vapor sorption analysis of the precipitate obtained in the dissolution study.

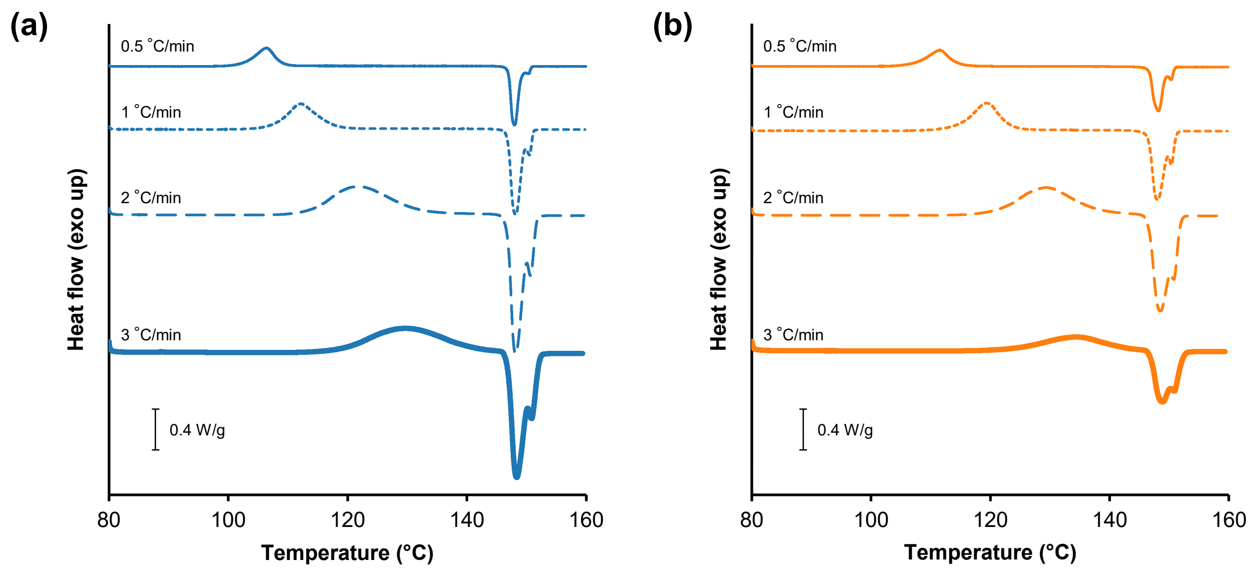

Figure S9. Representative DSC thermograms of amorphous TFD annealed at (a) −20 °C and (b) 25 °C for 40 days with various heating rates: 0.5 °C/min (solid line), 1 °C/min (dotted line), 2 °C/min (dashed line) and 3 °C/min (bold line).

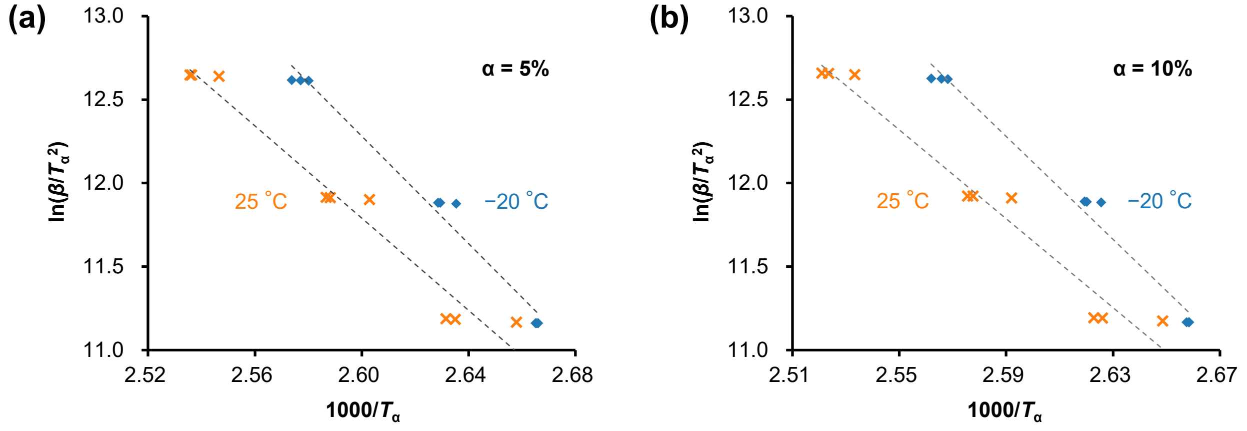

Figure S10. Kissinger-Akahira-Sunose plots for cold crystallization process of amorphous TFD annealed at −20 °C (blue diamond) and 25 °C (orange cross) for 40 days at isoconversion rate of (a) 5% and (b) 10%.

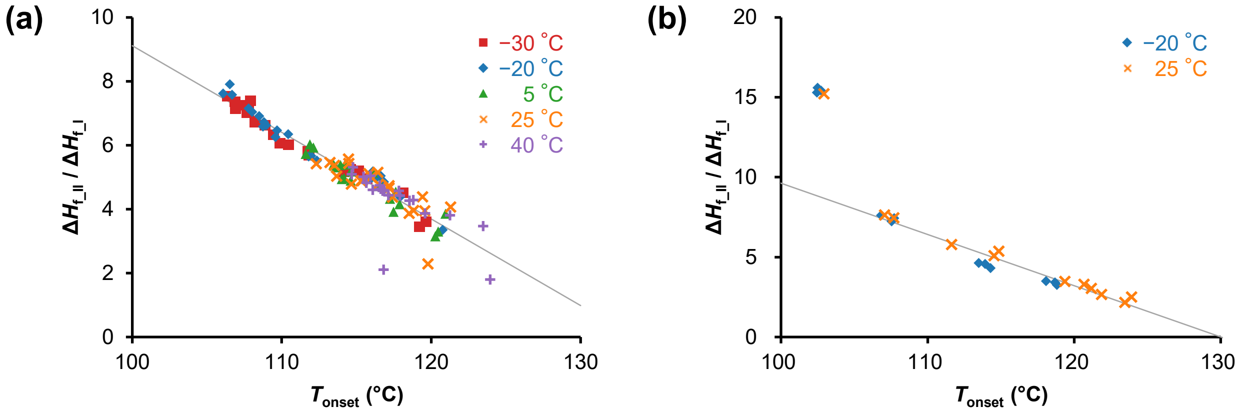

Figure S11. Relationship between T_{onset} and $\Delta H_{\mathrm{f\_II}}/\Delta H_{\mathrm{f\_I}}$ of amorphous TFD annealed at the temperatures indicated in the figure. (a) Samples were annealed for up to 40 days and heated at a rate of 1 °C/min. (b) Samples were annealed for 40 days and heated at rates of 0.5, 1, 2 and 3 °C/min.

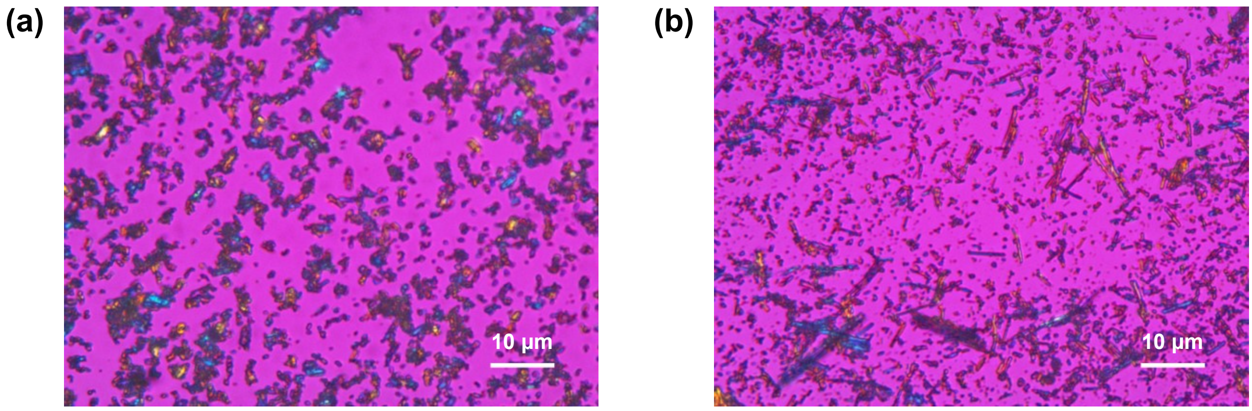

Figure S12. Polarized light microscopic images of (a) form I and (b) form II of TFD crystals.

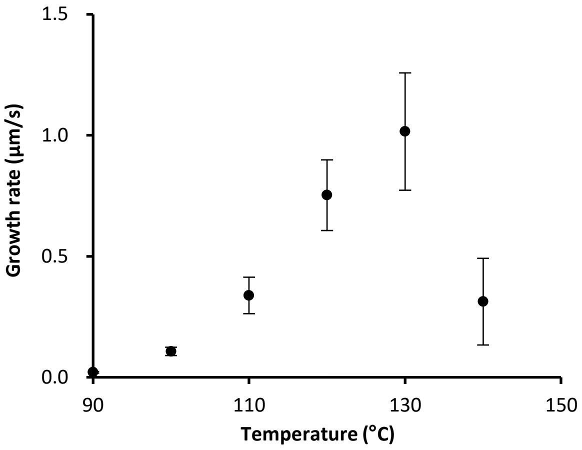

Figure S13. Temperature dependence of growth rate of form I of TFD crystals. Data are the average of at least three samples. Error bars indicate ± standard deviation.

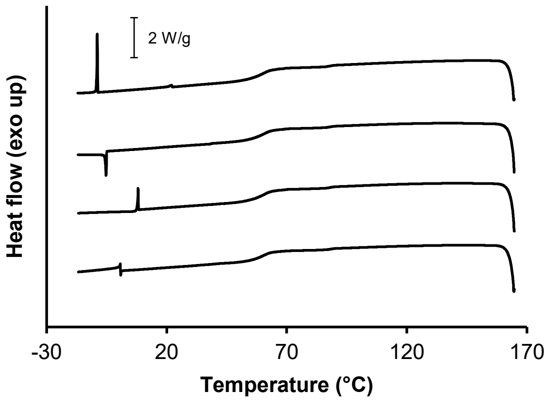

Figure S14. Representative DSC thermograms of TFD melt during cooling to −20 °C.

Microscopic observation of amorphous TFD in DSC pans

Amorphous TFD was prepared by the procedure described in **2.2 Preparation of amorphous TFD** without using the lid of the DSC pan. Microscopic observation was performed using an M205 C encoded stereo microscope (Leica Microsystems, Wetzlar, Germany) equipped with a MC170 HD microscope camera (Leica Microsystems). Annealed amorphous TFD was collected from the stability chamber after 7 days and illuminated with a fiber optical illuminator. Images were captured with LAS software (Leica Microsystems).

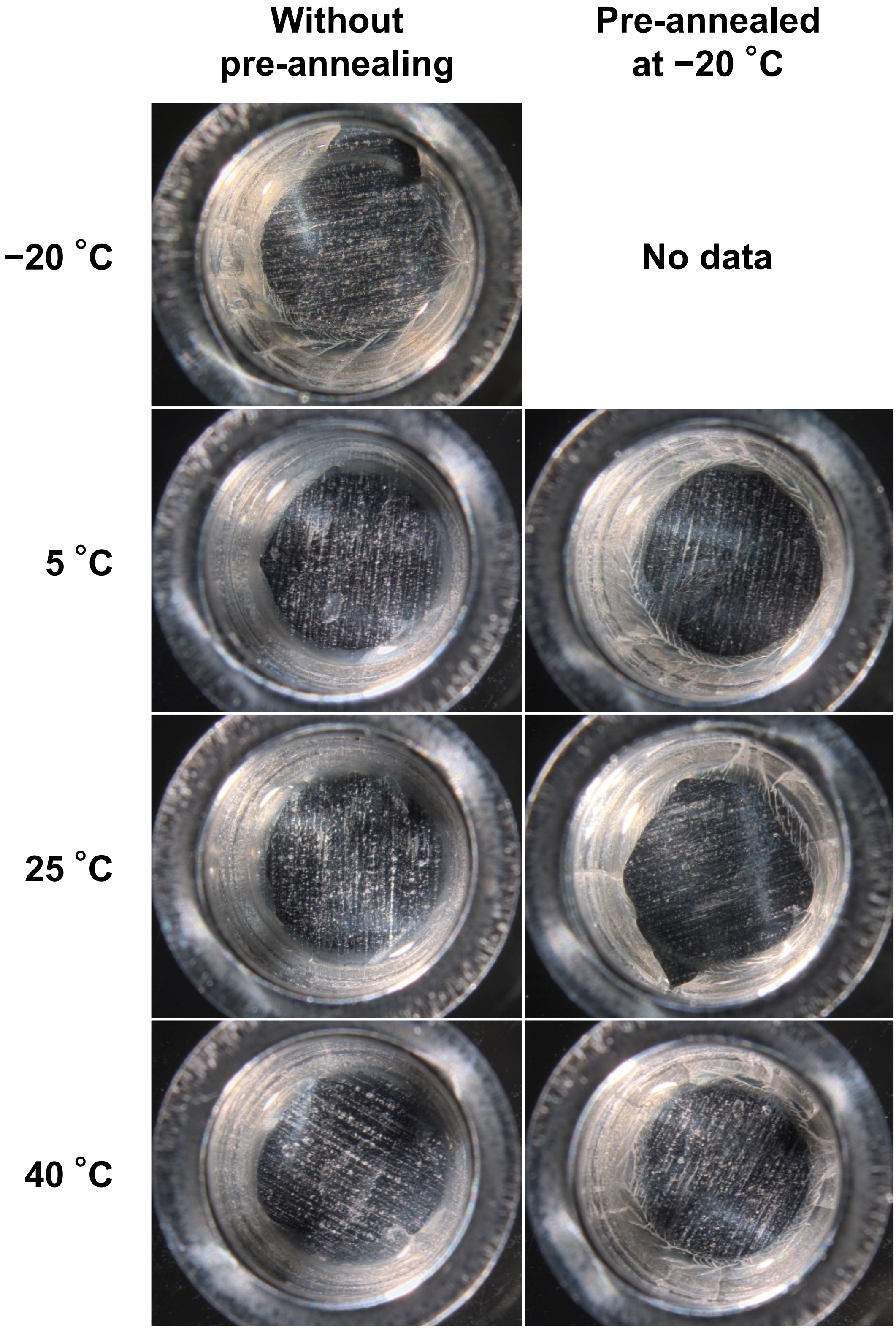

Figure S15. Representative microscopic images of amorphous TFD annealed at the temperatures indicated in the figure for 7 days. TFD melts were cooled to 25 °C (without pre-annealing) or cooled to −20 °C and held isothermal for 10 min (pre-annealed at −20 °C) before annealing at the target temperatures. Images are representative of at least three samples for each condition.

TGA of amorphous TFD

Amorphous TFD was prepared by the procedure described in **2.2 Preparation of amorphous TFD**. TGA was performed using a TGA 5500 under a nitrogen atmosphere with a flow rate of 25 mL/min. A DSC pan containing annealed amorphous TFD was collected from the stability chamber after 7 days and placed on a platinum sample pan for TGA. Samples were heated from room temperature to 250 °C at a rate of 5 °C/min. Weight (%) was calculated according to the following equation:

$$\text{Weight (\%)}(T) = \frac{\text{Weight measured by TGA}(T) - \text{Weight of DSC pan and lid}}{\text{Weight of TFD}} \times 100$$

where weight of DSC pan and lid and weight of TFD were as measured at the time of preparation of amorphous TFD.

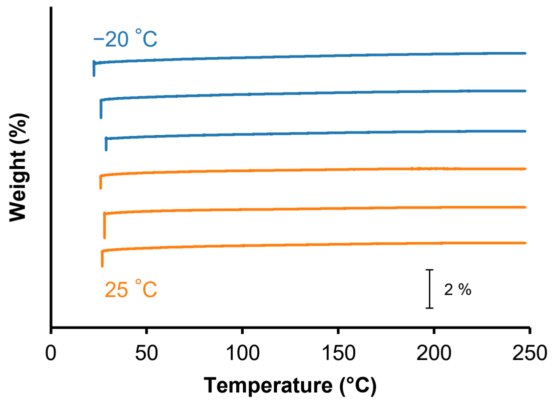

Figure S16. TGA thermograms of amorphous TFD annealed at −20 °C (blue) and 25 °C (orange) for 7 days. Average and standard deviation of weight (%) just after the start of heating was 100.01 ± 0.01.
